# Conscientiousness, Students’ Goal Orientation, and Reasoning Ability: Significance for Educational Standards

**DOI:** 10.3390/jintelligence12010011

**Published:** 2024-01-22

**Authors:** Selina Weiss, Martin Böhnisch

**Affiliations:** 1 Department Individual Differences and Psychological Assessment, Institute of Psychology and Education, Ulm University, 89081 Ulm, Germany; 2 Bühl-Realschule Dornstadt, 89081 Ulm, Germany; martin.boehnisch@rsd-intranet.de

**Keywords:** personality, intelligence, goal orientation, academic performance

## Abstract

Previous studies show that students’ goal orientation and conscientiousness are related to academic performance. Few studies, however, allow conclusions to be drawn about the factor structure of goal orientation and its distinctions from conscientiousness. In a study with *N* = 145 secondary school students (*M* = 13.9, *SD* = 0.85; 41% male), we investigated if the residuals of latent factors of goal orientation are still meaningfully correlated with academic performance and reasoning. Based on structural equation models, we have replicated the theoretically derived four-factor structure and showed that conscientiousness explains 29% of the variance in learning goals and 40% of the variance in work avoidance. Furthermore, we show that the residuals of goal orientation are mainly not significantly related to reasoning or educational standards (only work avoidance correlated with reasoning, and performance goals correlated with educational standards). Educational standards were highly correlated with reasoning. Implications for school practice and possible interventions are discussed.

## 1. Introduction

In the literature, cognitive abilities are often described as the most critical predictor of school performance (Kuncel et al. 2001). Furthermore, meta-analytical findings support the importance of cognitive abilities for scholastic achievement, especially in scientific school subjects such as mathematics and languages (Roth et al. 2015). However, more factors are deemed essential for academic achievements, such as conscientiousness or goal orientation (e.g., Steinmayr et al. 2011). In the study at hand, we aim to replicate the proposed factor structure of a German measure of goal orientation using confirmatory factor analysis. Next, we examine the distinction of goal orientation from conscientiousness and its correlation with reasoning ability and educational standards in a cohort of secondary school students (*Realschule*).

### 1.1. Defining Students’ Goal Orientation

In the educational literature, the achievement goal theory has included two facets when it comes to the study of motivation: achievement goals and goal structures (Bardach et al. 2020). Achievement goals include so-called personal factors of the students themselves (e.g., students aim to learn), while goal structures describe any contextual factors, such as teachers’ instructions (Bardach et al. 2020). The achievement goals (personal factors or facets) focus on the student’s aim to enroll and engage in a variety of learning activities (Meece et al. 2006). Meta-analytical findings imply that these two facets (achievement goals and goal structures) are highly correlated (Bardach et al. 2020). Our study focuses on personal factors based on the analysis of achievement goals. To this end, we include four different personal factors (e.g., Elliot and Church 1997; Nicholls 1984; Steinmayr et al. 2011): goals regarding learning, performance avoidance, performance approach, and work avoidance. Learning goals focus on striving to increase one’s own competencies. Performance approaching is characterized by the will to demonstrate competence, while performance avoidance includes the seeking, not to show any lack of competencies. On the other hand, work avoidance is based on the idea of working as little as possible. In previous studies, learning goals were positively correlated with performance approach (e.g., *r* = 0.30; Steinmayr et al. 2011) and negatively with work avoidance (e.g., *r* = −0.30; Steinmayr et al. 2011). For example, the latter implies that the higher the striving for competencies, the lower the work avoidance. Higher learning goals are related to higher achievement (e.g., Greene and Miller 1996), while work avoidance is typically negatively associated with achievement (e.g., Spinath et al. 2002b). This relation makes intuitive sense. The two performance goals showed high correlations with one another (e.g., *r* = 0.55; Steinmayr and Spinath 2009). Such a high correlation on a manifest level and the theoretical closeness of the factors might indicate that these two factors are not statistically distinguishable. Other than that performance approach was only weakly related to work avoidance (e.g., *r* = 0.16; Steinmayr et al. 2011), and performance avoidance was moderately related to work avoidance (e.g., *r* = 0.44; Steinmayr et al. 2011). Previous studies mostly distinguish these four factors as independent factors related to one another (SELLMO; Spinath et al. 2002a). However, it should also be questioned if a common factor of goal orientation exists. In the literature, the proposed four-factor structure is mostly based on theoretical models and exploratory factor analysis. Still, previous publications lack a rigorous comparison of latent models that test for different factorial solutions. We argue that three different models can be compared against one another: (1) the established four-factor solution with four correlated factors, (2) a general factor of goal orientation that does not distinguish between different factors and would imply a total score, and (3) three factors of goal orientation with only one performance factor based on the high correlation of the two performance scales.

### 1.2. Relation with Personality Traits, Reasoning Ability, and Academic Achievement

#### 1.2.1. Personality Traits

Personality is mainly described by five personality traits: openness, conscientiousness, extraversion, agreeableness, and neuroticism (McCrae and Costa 1989). The five factors are assessed by self-reports that cover perfectionism (conscientiousness), openness to new experiences and intellect, sociability (extraversion), willingness to cooperate/thoughtfulness (agreeableness), and emotional vulnerability (neuroticism). These factors return to lexical analysis (e.g., Allport and Odbert 1936) and are well established in adults and children (Bleidorn and Ostendorf 2009; Ostendorf 1990). In general, the five traits have been related to school success (Neuenschwander et al. 2013) and social problems, anxiety, depression, and hyperactivity (e.g., De Pauw and Mervielde 2010; Van den Akker et al. 2010). In particular, the factor of conscientiousness is often deemed necessary for school and academic success and has even been related to academic performance independently from cognitive abilities (e.g., Poropat 2009). Therefore, one question is how close are goal orientation traits to conscientiousness? The existence of jangle fallacies, implying that two scales are equivalent but just named differently (Kelley 1927) has been studied a lot in the past years due to the frequent publication of so-called new constructs (see, for example, grit and conscientiousness, Credé et al. 2017; or self-compassion and conscientiousness, Pfattheicher et al. 2017). Based on the recently published semantic scale network, we can identify scales that show a semantic similarity to each item (Rosenbusch et al. 2020). When it comes to the goal orientation items that were used in this study, the most apparent semantic similarities (>0.50; Landauer et al. 1998) are between learning goal items and other scales that capture school motivation (e.g., Hickey et al. 2001; Skaalvik and Rankin 1995; Zimmerman et al. 1992). Due to the number of scales that assess motivation, goals, efficacy, etc., the closest neighbors (of the items used in this paper) that are provided by the shiny app of Rosenbusch et al. (2020) are primarily from these areas. However, comparing the items of work avoidance with conscientiousness items (see Table 1 for the translated items used in this study) makes it evident that reversed conscientiousness (e.g., being lazy, being less diligent, unwilling to do things that are set out to be completed), is content-wise similar to contents captured in work avoidance (e.g., keeping the workload low, not working hard).

Previous correlations on the manifest level, however, give a first hint that the goal orientation factors appear to be distinct from conscientiousness (*r_learning goals_* = 0.34; *r_performance approach_* = 0.22; *r_work avoidance_* = −0.34; Steinmayr et al. 2011). Nevertheless, the literature lacks an examination of this relation on the latent level, examining the incremental validity of these factors beyond conscientiousness (Steinmayr et al. 2011). Moreover, the other personality traits seem relatively remote from the contents of the goal orientation scales. Agreeableness shows a weak but significant correlation with all goal orientation factors, while extraversion, openness, and neuroticism are only sporadically and very small related to goal orientation (Steinmayr et al. 2011).

#### 1.2.2. Reasoning Ability and Academic Achievement

Previous research on cognitive abilities has described a number of broad factors that can be found under a general factor of cognitive abilities (e.g., Carroll 1993). One of the most prominent factors is fluid intelligence (reasoning ability; Wilhelm 2005). The literature shows that cognitive abilities—such as reasoning ability—are the most critical predictors of school success and academic achievement (e.g., Gottschling et al. 2012). Academic achievement is often indicated by grades (GPA). However, several national assessments and comparative studies of educational standards can also serve as indicators for academic achievements (e.g., VERA-8, Schult and Wagner 2019).

Cognitive abilities, such as reasoning ability or fluid intelligence, have not only been identified as an important predictor of school success but these abilities have also been shown to be linked to the accumulation of knowledge in the context of the investment theory of cognitive abilities (e.g., Cattell 1963). Thereby, fluid abilities stand in strong interrelation with other non-cognitive investment traits, such as openness and need for cognition (Von Stumm and Ackerman 2013; Ziegler et al. 2012). Even though motivation and student achievement can also be understood as investment traits, more recent reviews exclude such goal-striving traits as these traits focus on finding and sticking to learning opportunities (Von Stumm and Ackerman 2013). Therefore, it can be asked how such goal-striving traits are related to cognitive abilities, such as reasoning ability, on the one hand, and academic achievement, on the other. The literature has shown that scholastic interest or the academic self-concept has also been associated with higher academic achievement even beyond the effects of cognitive abilities on academic achievement (Jansen et al. 2016; Jansen et al. 2014). Even though scholastic interest is a different construct (compared to students’ goal orientation), motivation and learning goals were also found to predict PISA test achievement beyond intelligence (Kriegbaum et al. 2015; Steinmayr et al. 2011). Other than that, goals are mediational between the students’ motivation and achievement (Dickhäuser et al. 2016). GPA has also been related to students’ goals, further stressing the importance of students’ goals in academic achievement. However, the relationship between different factors of students’ goal orientation and cognitive ability requires further investigation since only work avoidance was significantly correlated with different measures of intelligence previously (Steinmayr and Spinath 2009; Steinmayr et al. 2011).

### 1.3. Research Aims

This study’s first aim is to investigate the factor structure of the student’s goal orientation by applying confirmatory factor analysis to assess the scale’s factor structure and construct validity. Based on previous findings and theoretical considerations, we expect to find four factors that are significantly related to one another: learning goals, performance approach, performance-avoidance goals, and work avoidance.

Second, we aim to examine if the measurement model we establish based on the research aim one is distinct from the personality factor conscientiousness. We argue that especially learning goal orientation and work avoidance should be strongly predicted by conscientiousness and might not be distinct from the conscientiousness factor, implying a jangle fallacy. If the factors are distinct from conscientiousness, we expect their link to be below unity and the residuals of the latent factors of students’ goal orientation to be meaningfully related to one another or other covariates.

Third, based on a larger structural equation model, we investigate if the residuals of students’ goal orientation are correlated to academic performance in terms of eighth-class educational standards (VERA-8; Schult and Wagner 2019) and reasoning ability. We expect that reasoning ability is highly correlated with students’ academic performance. Other than that, we expect that the residuals of the student’s goal orientation are only weakly or not significantly correlated with academic performance and reasoning ability.

## 2. Materials and Methods

The following sections provide information on the study design, sample, and measures. The data and scripts that can be used to reproduce all analyses can be found online in the OSF repository [https://osf.io/8yfaw/]. The research project is not covered by the Declaration of Helsinki on research involving human subjects and, therefore, in the opinion of the Ethics Committee, does not require an ethics vote. The study does not collect sensitive data on the health or sexuality of the participants.

### 2.1. Sample

The participants were recruited in all eighth and ninth grades at a secondary school [*Realschule*] in Baden Wuerttemberg. The sample included *N* = 145 participants, with *n* = 74 students in eighth grade and *n* = 71 in ninth grade. Students were only allowed to participate if written parental informed consent was provided. The participation was voluntary within a school lesson and not related to any school assessment or evaluation. The students’ ages ranged from 13 to 18 years (*M* = 13.9; *SD* = 0.85), and 41% of the students reported being male.

### 2.2. Procedure and Design

This study was administered within a school lesson (45 min) on tablets using the programming software Inquisit (6) (Millisecond 2022) and on paper and pencil. The assessment of the student’s goal orientation (Spinath et al. 2002a), personality (Kupper et al. 2019), and reasoning ability (Wilhelm et al. 2014) were administered on tablets. Further administered tests were not subject to this manuscript and are therefore not reported. Due to the scope of the study, only goal orientation, personality factors, and reasoning ability are included in this paper. The results of the already gathered VERA-8 tests (eighth-grade comparative study in German schools) were later merged with the results of the ninth-grade testing.

### 2.3. Measures

In the next section, we present all questionnaire measures and the measures for students’ abilities and performances.

#### 2.3.1. Students’ Goal Orientation

The student’s goal orientation was assessed by a German self-report scale that includes four subscales in order to assess the different school-related goals (“Skalen zur Erfassung der Lern- und Leistungsmotivation”, SELLMO; Spinath et al. 2002a). The SELLMO includes the assessment of learning goals (e.g., “In school, I need to learn as much as possible.”) based on eight items; the performance approach (e.g., “In school, it is important to me that others think I am smart.”) based on seven items; the performance-avoidance goals (e.g., “In school, it is important to me not to give wrong answers to questions of the teacher.”) based on eight items; and work avoidance (e.g., “In school, it is important to me to do as little work as possible.”) based on eight items. The scales use a 5-point Likert scale indicating the students’ agreement.

#### 2.3.2. Personality

The personality was assessed using a German short version of the Big-five Inventory for Children and Adolescents (BFI-K KJ; Kupper et al. 2019). The BFI-K KJ includes the big five factors (conscientiousness, extraversion, agreeableness, neuroticism, and openness) based on 26 Likert-scale items. For the purpose of this paper, only the factor of *conscientiousness* (see Table 1 for translated items) was considered for the analysis. Compared to the results reported by Kupper et al. (2019), we found a higher conscientiousness on the scale level (*M* = 19.39, *SD* = 3.97). The correlations of the personality scales were similar to those reported by Kupper et al. (2019).

#### 2.3.3. Reasoning Ability

The student’s reasoning ability was assessed based on a figural reasoning test for the eighth to tenth grades (Wilhelm et al. 2014). The figural reasoning scale was composed of a sequence of geometric drawings (so-called Charkow tasks) that changed their shading and form according to certain rules. Within a figural sequence, the student is asked to decide which of two figures out of three options completes the series. The figural reasoning scale included 16 items (presented with increasing difficulty) with a 14 min time limit. In order to receive credit for a correct item, the student has to select both figures correctly. Out of the 16 items, the students solved *M* = 7.32 items (*SD* = 2.56). In the results, we used four parcels to model reasoning ability that were randomly generated.

#### 2.3.4. VERA-8

The eighth-grade comparative study in German schools (VERA-8, Schult and Wagner 2019) tests several areas of competencies in different subjects. The results of VERA-8 refer to the educational standards of middle schools. The feedback includes the boundaries of educational standards and the mean achievements of the class, the school, and all schools in the states. In the analysis, we included the previous educational standards of ninth-grade students. The educational standards are divided into six areas: standard 1a/1b corresponds to the lower minimum standard, standard two corresponds to the minimum standards, standards three and four correspond to normal standard and normal standard plus, and standard five is the optimal educational standard. These standards were evaluated for mathematics, German orthography, German reading ability, English listening comprehension, and English reading ability. None of the students reached the optimal standard in mathematics, and 24% only reached the lower minimum standards. The optimal standards were reached in English and German; fewer students showed lower minimum standards (e.g., English reading ability, 12%; English listening comprehension, 3%).

### 2.4. Statistical Analyses

We computed several measurement models that were later used within a larger structural equation model. For evaluating the fit of all models, we used the comparative fit index (CFI), the root mean square error of approximation (RMSEA), and the standardized root mean square residual (SRMR) (Hu and Bentler 1999). Applying these fit indices, a CFI ≥ 0.95, RMSEA ≤ 0.06, and SRMR ≤ 0.08 indicate a very good fit. However, fit indices above CFI > 0.90 and RMSEA < 0.07 can be deemed acceptable. The statistical analysis is based on *R Studio* software using mostly the packages *lavaan* (Rosseel 2012) for all latent variable models and *psych* (Revelle 2018) for the outlier analysis and further descriptive statistics. All models were estimated with a *robust maximum likelihood* (MLR) estimator. Due to the different scales (e.g., reasoning, educational standards, Likert scales) that were used in the data, all scores were z-standardized for structural equation modeling.

In the following, we report measurement models for the variables of interest. As the modeling of the student’s goal orientation is a part of the research aims, this will be described in the results section. The personality factor *conscientiousness* was modeled using three parcels. Therefore, the model was exactly identified. The conscientiousness factor reached good reliability ω = 0.76. The reasoning ability was modeled based on four randomly generated parcels out of the 16 items. The model fitted the data well (χ^2^_(2)_ = 1.71; *p* = 0.43; CFI = 1.00; RMSEA = 0.00; SRMR = 0.02). A factor displaying reasoning ability reached acceptable reliability ω = 0.65.

**Figure 1:**
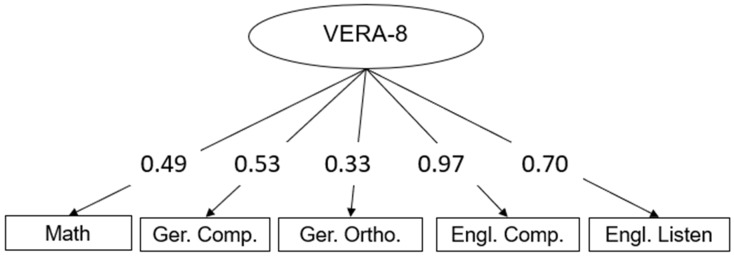
Schematic measurement model of VERA-8. Note. Ger. Comp = German reading ability, Ger. Ortho. = German orthography, Engl. Comp = English reading ability, Engl. Listen = English listening comprehension.

The results of the VERA-8 were modeled as a one-factor model displaying the general educational standards reached in the eighth class. The one-factor model fitted the data well (χ^2^_(5)_ = 7.22; *p* = 0.21; CFI = 0.97; RMSEA = 0.08; SRMR = 0.07) and showed good reliability ω = 0.82. The factor loadings were all significant with the highest factor loading in English reading comprehension. The model is schematically displayed in Figure 1. It has to be noted that the VERA-8 data were only available for ninth graders who have already completed VERA-8 in the last year. Therefore, the model was based on *n* = 68 observations while the missing values were estimated using the *full information ML* estimator (Schafer and Graham 2002). This implies that missing covariances and variances on the sample level were estimated so that the model estimation was based on *N* = 145 persons.

**Figure 2:**
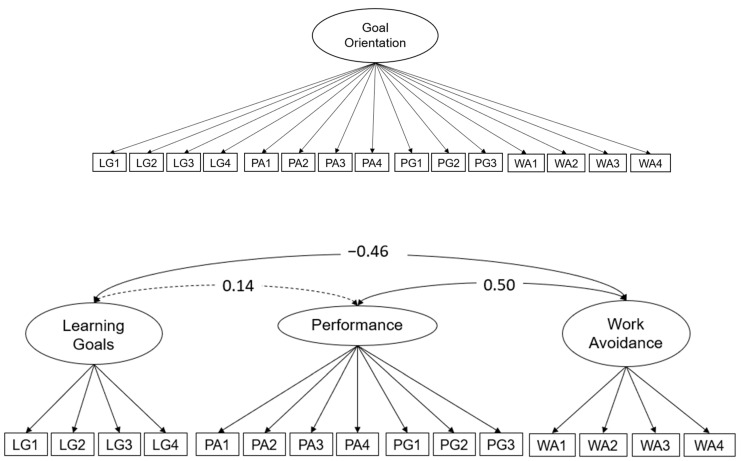

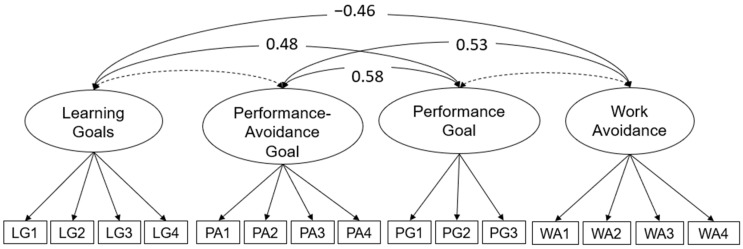
*Schematic models of students’ goal orientation. Note:* dotted paths display non-significant relations.

**Figure 3:**
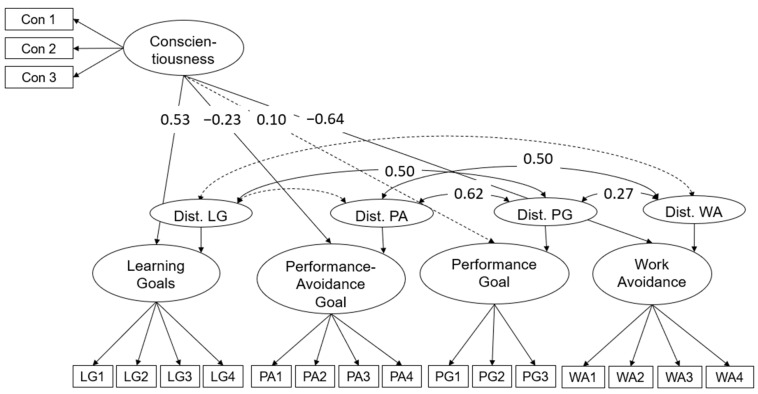
*Students’ goal orientation predicted by conscientiousness. Note:* dotted paths display non-significant relations. Dist. = residuals of the according latent factors.

**Figure 4:**
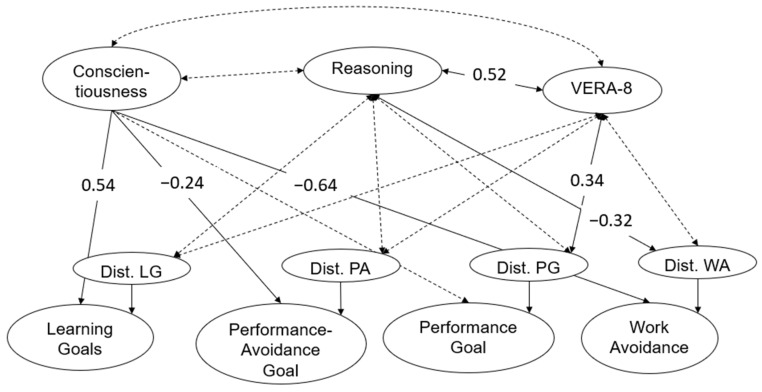
*Schematic model of students’ goal orientation predicted by conscientiousness, correlated with VERA-8. Note:* dotted paths display non-significant relations. The correlations between the residuals of the four latent factors of students’ goal orientation are estimated but not displayed for readability of the figure (see Figure 3 for the correlations).

Given the limited sample size that was used in our study, we used a post hoc sample size determination to estimate the necessary power to reject specific models. We used semPower (Jobst et al. 2023) in order to test for the required sample size that is needed to reject a one-factor model (see Figure 2) if the population model includes four correlated factors. The correlations for the four correlated factors were used from Steinmayr et al. (2011) (see Table 1 in Steinmayr et al. 2011). Even though larger samples are always encouraged in order to reduce error risks (Moshagen and Erdfelder 2016), we found that a sample of only *N* = 30 observations yields enough power (>0.80, α = 0.01) to make such a model comparison (please note that in order to be more conservative, we have set the α error to 0.01). Although the power for the model comparison would be enough at this sample size, this sample size would be insufficient for model convergence and reliable parameter estimates. As described by Wolf et al. (2013), the sample size requirements depend on a variety of model parameters (number of factors and indicators, loadings, missingness, etc.). In the model described in Figure 3, the mean loading was λ = 0.73 and in Figure 4, the mean loading was λ = 0.69. In their sample size estimation, Wolf et al. (2013) describe that given a 3-factorial solution with 3 per factor indicators, correlations of 0.30, and loadings of 0.65, the power value is 0.71 at *N* = 140. Given a 3-factorial solution with 6 indicators per factor, the power at *N* = 140 is 0.82. The detailed power analysis with more information can be found in the online repository [https://osf.io/8yfaw/].

## 3. Results

### 3.1. First Research Aim: Factor Structure of Students’ Goal Orientation

The literature proposes that the student’s goal orientation includes four scales. On a manifest level, we replicated correlations in the same height and direction as previously found in the literature (see Table 2). As expected, work avoidance is negatively related to the student’s learning goals.

In order to investigate the factor structure of the student’s goal orientation, we tested several factor models using randomly generated parcels within each scale, as models on the item level did not reach good fit indices.

We compared three different models that are schematically displayed in Figure 2. The first model indicated a one-factor solution that would statistically justify a total score of students’ goal orientation. This model did not show any acceptable model fit (χ^2^_(90)_ = 497.34; *p* = 0.00; CFI = 0.47; RMSEA = 0.18; SRMR = 0.18). Based on the high correlations on a manifest level between the theoretically derived factors of performance avoidance and performance goals, we next tested a solution with three correlated factors. This model fitted the data better but did also not reach an acceptable model fit (χ^2^_(105)_ = 192.44; *p* = 0.00; CFI = 0.86; RMSEA = 0.09; SRMR = 0.10). Lastly, we tested a model using four correlated factors (see Figure 2). This correlated factor model fitted the data best and reached fit indices implying a good fit (χ^2^_(84)_ = 99.99; *p* = 0.11; CFI = 0.98; RMSEA = 0.04; SRMR = 0.05). All loadings were statistically significant, and λ > 0.61. The correlations between the factors are displayed in Figure 2. The reliability of the factors was acceptable: ω_LG_ = 0.75, ω_PA_ = 0.87, ω_PG_ = 0.77, and ω_WA_ = 0.84.

### 3.2. Second Research Aim: Distinctiveness from Conscientiousness

Second, we investigated if the factors displaying learning goals and work avoidance are distinct from the personality factor conscientiousness. Therefore, the correlated factor model found in research aim one was used, and the four factors were predicted by conscientiousness (see Figure 3). This model fitted the data well (χ^2^_(125)_ = 152.99; *p* = 0.05; CFI = 0.97; RMSEA = 0.04; SRMR = 0.06). As expected, conscientiousness did not significantly predict performance goals and only weakly predicted performance avoidance (*p* = 0.04). Next, conscientiousness explained 29% of the variance in learning goals and 40% of the variance in work avoidance. In addition, we allowed the residuals to correlate with one another. The residuals of the latent factors learning goals and work avoidance were not correlating anymore (*r* = −0.18; *p* = 0.24). Also, the correlation between the residual of the factor work avoidance and the latent factor performance goal was lower than found in the model displayed in Figure 2 (*r* = 0.27; *p* = 0.03).

### 3.3. Third Research Aim: Goal Orientation, Conscientiousness, Reasoning Ability, and Educational Standards

Showing that the student’s goal orientation cannot be fully explained by conscientiousness, we next tested how the residuals of these factors are related to educational standards and reasoning. Therefore, the model in Figure 3 was expanded by the results of VERA-8 (displaying educational standards in eighth grade) and the latent variable for reasoning ability. This structural model is schematically displayed in Figure 4. The model fitted the data well (χ^2^_(303)_ = 412.94; *p* = 0.00; CFI = 0.91; RMSEA = 0.05; SRMR = 0.08). We found that conscientiousness and VERA-8 were not correlated, as well as conscientiousness and reasoning. In addition, only the residual of the latent factor performance goal was correlated with the educational standards (*r* = 0.34; *p* = 0.03). And only the residual of the latent factor work avoidance was correlated significantly negatively with reasoning (*r* = −0.32; *p* = 0.03).

## 4. Discussion

### 4.1. Summary of the Results

With this study, we examined three research aims. First, we tested three competing models in order to examine the factor structure of students’ goal orientation. We found four correlated factors best displayed the construct (e.g., Spinath et al. 2012). A solution including a general factor of students’ goal orientation or three correlated factors (only one performance factor) fitted the data significantly worse. This finding implies that students’ goal orientation—as measured here—comprises four factors with acceptable reliability that can be distinguished from another: learning goals, performance approach, performance avoidance, and work avoidance. As expected, learning goals and work avoidance were negatively related, while all other factors were positively correlated with one another (Steinmayr et al. 2011). Second, we used this model in order to assess if these four factors are distinct from the personality trait conscientiousness. Conscientiousness explained significant variance in learning goals (29%) and work avoidance (40%). However, the amount of explained variance indicated that these factors capture something above and beyond conscientiousness. Interestingly, the correlation between the residuals showed that the residuals of the factor learning goals and work avoidance were not correlated anymore, after adding conscientiousness to the model. This indicates that the correlation between the two, which is often reported in the literature (e.g., Steinmayr et al. 2011), is driven by conscientiousness. Third, we enlarged this model by adding reasoning ability (fluid intelligence) and educational standards to the model. Conscientiousness was not correlated to either of them. As expected, logical reasoning and educational standards were highly correlated. Furthermore, logical reasoning was negatively correlated with residual work avoidance, implying that the higher the reasoning ability, the less the work avoidance—after controlling for conscientiousness. The educational standards in terms of VERA-8 were only significantly correlated with the residual of the performance approach. This indicates that—after controlling for conscientiousness—the performance approach as characterized by the will to demonstrate competence is correlated with the VERA-8, which means that the higher the educational standards reached, the higher the student’s intention to demonstrate their competencies.

### 4.2. Implications for Schools

This study’s findings are significant for school practice on three levels: (a) the individual level, (b) the collective level, and (c) the institutional level. In the following, we define these three levels before we discuss their practical relevance.

The individual level can be understood as a teacher’s counseling of parents and students and might include aspects such as tutoring, homework support, etc. It plays a crucial role as it precedes any other actions at the individual level. Depending on the occasion, counseling for parents (e.g., at their request) may be about performance, behavior, self-organization, homework, or other matters. Students receive counseling in the form of feedback on their performance. Referring to the paper at hand, the results of VERA-8 (German, mathematics, English; Schult and Wagner 2019), for example, should be discussed at an individual level (the parents and the class receive feedback, according to the Verwaltungsvorschrift des Kultusministeriums zu den zentral angefertigten Lernstandserhebungen 32–6500.4/559/9 2016).

The collective level includes promoting groups of students in a competence area. Subject-specific and cross-curricular competencies can be promoted. The promotion of subject-specific competencies primarily concerns the subjects of German, English, and mathematics. Cross-curricular competencies include learning competencies, social competencies, and problem-solving skills (Klieme et al. 2014). The student councils are responsible for organizing the promotion of subject-specific competencies. A teacher is responsible for the support and bases the choice of methods and materials on the student performance or the results of a diagnosis (e.g., *Hamburger Schreibprobe* (May 1997), etc.). Whether a student belongs to a support group or not depends on several factors: the approval of the class conference, the parents’ consent, student’s performance ((Kinder und Jugendliche mit besonderem Förderbedarf und Behinderungen, IV/1-6500.333/61 1999), e.g., special needs in reading) and diagnostic tests. Concerning the results of VERA-8, the formation of support groups is not planned but would be possible if the school had the corresponding hours available.

The institutional level concerns the creation of structures for individual and collective support of students. For example, collective support can occur during regular lessons, parallel to them, or in special marginal lessons.

In the following sections, we discuss the relevance of the results of this study on different levels. We aim to answer the following questions: Are the findings relevant to the according level? What measures could complement counseling and support practice? Which findings are relevant for pedagogical practice at which level? Which measures could be supplemented, expanded, or replaced to promote students’ abilities more effectively and efficiently?

Before answering these questions, the terminology applied in this paper must be related to the conventionally applied terminology in school contexts. In schools, reasoning is often understood as a competency that has to be acquired domain specifically (Baumert et al. 2007). However, we instead understand reasoning as measured in the study at hand as a general intellectual ability (Weinert 2002). There is little distinction between learning and performance goals since classwork and tests require both and are sanctioned. Conscientiousness is described, if at all, as a cross-curricular skill and falls more under the realm of education or the school’s educational mission. Despite these conceptual differences, there are several commonalities between terminology as applied here and in schools. It is well acknowledged that academic achievement, such as that measured by VERA-8, is related to the students’ general intellectual abilities (Weinert 2002).

Furthermore, school performance is interpreted at all three levels. Therefore, it is advantageous for school administrators, subject committees, and teachers to have more information about the background of school learning. Learning assessments are the first step here. But only the correlation with other skills (e.g., goal orientation, conscientiousness) helps the teacher to have a more accurate picture of the student or the class. The results of the present study show a connection between the educational mandate (e.g., learning objectives) and the educational mandate (e.g., conscientiousness). That VERA correlates more with achievement goals and less with learning goals may be surprising; however, it is due to the nature of the study, as VERA takes place once. Even though VERA is not graded, it is about performance compared to others.

In the following, consequences for school practice are formulated for all three levels. These focus on the implementation of goal orientation (first research aim), conscientiousness (second research aim), and the interplay of goal orientation, conscientiousness, cognitive abilities, and educational standards (third research aim).

#### 4.2.1. Implementations on the Individual Level

The distinction between learning and performance goals and work- and performance-avoiding behavior is essential for counseling. Poor performance can have various causes: cognitive, voluntary, or motivational (Campbell et al. 1993). But the reason may also lie in the absence of learning goals. Providing a different interpretation of school performance is a first step. A second step would be to focus on establishing learning goals. Köller (1998) showed that students with solid learning goals demonstrate more remarkable school achievements (Wild et al. 2001). Fostering learning goals—for example, in counseling—can be achieved by communicating grading and evaluations, providing the material conducive to learning (e.g., providing learning environments and systematic materials), offering the experience of autonomy, and communicating expectations. In sum, counseling should address quantitative aspects of learning (homework, exercise programs, more preparation for classwork) and qualitative ones (the main point of spelling, sentence structure, ecology, fractions, and what are good exercises). Making learning goals explicit is not just a requirement that can be placed on guidance; it also applies to instruction. As Hattie (2014) has shown in his research, this provides evidence that student learning can be significantly enhanced by instruction that is clear, well structured, and prioritizes important concepts. He also emphasizes that effective instruction should be responsive to students’ needs and provide active engagement and feedback opportunities.

#### 4.2.2. Implementations on a Collective Level

The results show that a substantial relationship exists between reasoning ability and subject-specific competencies (e.g., German, mathematics, and English). Indeed, reasoning ability cannot be influenced. However, if the teachers are aware of this fact, they can foster other student traits or trainable skills, such as argumentative skills. Subjects such as German, mathematics, and English would equally benefit from such programs. In addition, remedial subjects can focus more on learning goals and less on performance goals. This also fits in with the self-image of remedial teaching, in which grades are not usually given. Through learning plans, students can set their own goals during remedial instruction and designate areas of focus in which they have deficits. Future studies can then show if the residuals of learning goals are significantly related to achievements in remedial subjects.

#### 4.2.3. Implementations on Institutional Level

The legal requirements of the German states include the implementation of performance studies. The interpretation of the results and the derivation of appropriate measures are associated with this (Verwaltungsvorschrift des Kultusministeriums zu den zentral angefertigten Lernstandserhebungen 32–6500.4/559/9 2016). As the results of the study show, VERA-8 is mainly correlated with cognitive skills (such as reasoning) but also with the residual performance goals, which makes sense as the performance-approaching goals are defined by the will to show competence. These findings should be accounted for in presenting the results of VERA-8 at parent–teacher conferences. Reasoning ability and the willingness to demonstrate performance are essential in all core subjects, such as German, mathematics, and English.

### 4.3. Limitations of the Study

Although this study included various measures that allow analysis of constructs within their nomological net, several limitations must be considered when interpreting the results.

First of all, the results have to be replicated in larger samples. Given the model’s complexity, the sample size is very limited, and even though a post hoc power analysis yields enough power to reject specific models, a greater sample size is always encouraged to reduce any risk of errors (Moshagen and Erdfelder 2016). Furthermore, the sample of this study is limited to students from one school (Realschule) in one German state. This challenges generalizability. However, we found similar results regarding the factor structure and correlations between factors than were reported in previous research that included other school forms (Gymnasium), age of students, and German states (e.g., Steinmayr et al. 2011). Nevertheless, these results should be replicated in broader samples regarding the school types, states, and students’ ages.

Second, as in many studies, the task selection is debatable. In order to measure cognitive abilities, we only applied a measure of figural reasoning ability. Although fluid intelligence is known as the best indicator of general cognitive abilities (c.f. Wilhelm 2005), and figural tasks are the best indicators of reasoning ability (Wilhelm 2005), a broader assessment of cognitive abilities that includes further ability factors, such as crystallized intelligence or cognitive speed and creativity (Wilhelm and Kyllonen 2021) would be preferable. Furthermore, the assessment could be broadened toward a more comprehensive understanding of goals. For example, even though previous studies showed a high correlation between personal and contextual goal features, it would also be interesting to include such contextual goal structures in a broader sample (e.g., Bardach et al. 2020).

## 5. Conclusions

In the present study, we have examined the structure of students’ goal orientation, as well as its significance—among personality and cognitive abilities—for educational standards. First and foremost, the study shows that the four derived factors that can be distinguished in the study of goal orientation are distinct from conscientiousness; however, they are only of little significance for educational standards. The findings include that the construct of students’ goal orientation is no jangle fallacy with respect to conscientiousness, but it is also only weakly and partly (residual of performance goals) correlated with educational standards in terms of VERA-8. This implies that if students reach specific educational standards is driven mainly by cognitive abilities (reasoning ability) and not by traits such as personality and goal orientation.

## Figures and Tables

**Table 1:** Items that are used to measure work avoidance (Spinath et al. 2002a) and conscientiousness (Kupper et al. 2019).

Items Work Avoidance	Items Conscientiousness
School is all about…	I…
not having difficult tests or papers.	carry out tasks properly.
not having to do any work at home.	am comfortable, prone to laziness [R].
not having to solve difficult questions or tasks.	am diligent and work quickly.
not working so hard.	do what I set out to do.
that the work is easy.	am rather untidy [R].
to avoid having to do time-consuming tasks myself.	am easily distracted [R].
to get through school with little work.	
to keep the workload low at all times.	

*Note.* The items are initially in German. R = reversed coded items.

**Table 2:** Descriptive Statistics and manifest level intercorrelation for students’ goal orientation.

Scale	Sum (*SD*)	PA	PG	WA
Learning Goals (LG)	30.47 (4.69)	0.34	0.09	−0.37
Performance Avoidance (PA)	22.68 (5.02)		0.59	0.13
Performance Goal (PG)	22.23 (6.30)			0.42
Work Avoidance (WA)	22.11 (6.52)			

*Note.* Correlations displayed in gray are not statistically significant (*p* > 0.05).

## Data Availability

The data and all required scripts are available at [https://osf.io/8yfaw/].
